# Prevalence of peripheral arterial disease in subjects with moderate cardiovascular risk: Italian results from the PANDORA study Data from PANDORA (Prevalence of peripheral Arterial disease in subjects with moderate CVD risk, with No overt vascular Diseases nor Diabetes mellitus)

**DOI:** 10.1186/1471-2261-11-59

**Published:** 2011-10-07

**Authors:** Guido Sanna, Donatella Alesso, Malek Mediati, Claudio Cimminiello, Claudio Borghi, Amalia Lucia Fazzari, Mario Mangrella

**Affiliations:** 1 METIS Scientific Association of Italian Family Doctors, Roma, Italy; 2 Department of Medicine, Vimercate Hospital, Italy; 3 Department of Internal Medicine, S.Orsola-Malpighi Hospital, Bologna University, Italy; 4 Department of Economics and Territory, University of Rome "Tor Vergata", Italy; 5 R&D Department, AstraZeneca SpA, Milan, Italy

**Keywords:** Peripheral vascular disease, ankle-brachial index, atherosclerosis, risk factors, prevention

## Abstract

**Background:**

The PANDORA study has recently examined the prevalence of low ankle brachial index (ABI) in subjects with moderate risk of cardiovascular disease. This sub-analysis of the PANDORA study examines the prevalence of asymptomatic peripheral arterial disease (PAD), as determined by ABI, in Italian subjects presenting with moderate cardiovascular risk, in the absence of diabetes or overt vascular disease.

**Methods:**

PANDORA is a non-interventional, cross-sectional study that was performed in 6 European countries, involving subjects with at least one cardiovascular (CV) risk factor. The primary objective was to evaluate the prevalence of asymptomatic PAD using ABI. For this post-hoc sub-analysis, data were extracted for subjects enrolled in Italy, comprising 51.5% (n = 5298) of subjects from the original PANDORA study. Secondary objectives were to establish the prevalence and treatment of CV risk factors.

**Results:**

The mean age was 63.9 years and 22.9% (95% CI 21.7-24.0) of subjects presented with asymptomatic PAD. A range of risk factors comprising smoking, hypertension, low HDL-cholesterol, family history of coronary heart disease and habit of moderate-high alcohol intake were significantly associated with asymptomatic PAD (p < 0.0001). Statin treatment had the lowest incidence in Italian subjects. Furthermore, patients treated with statins were significantly less likely to have asymptomatic PAD than those who were not (p = 0.0001).

**Conclusions:**

Asymptomatic PAD was highly prevalent in Italian subjects, the majority of whom were not candidates for ABI assessment according to current guidelines. Findings from this study suggest that these patients should be carefully examined in clinical practice and ABI measured so that therapeutic interventions known to decrease their CV risk may be offered.

**Trial registration number:**

ClinicalTrials.gov: NCT00689377

## Background

Individuals who are diagnosed with lower extremity peripheral arterial disease (PAD) are associated with a reduced quality of life, in addition to an increased risk of atherosclerosis, stroke, cardiovascular (CV) morbidity and mortality [1-3]. Unfortunately the CV burden associated with the presence of PAD is similar, regardless of whether it is the asymptomatic or symptomatic form of PAD [4,5]. For this reason, current guidelines recommend the measurement of ankle-brachial index (ABI) to identify asymptomatic patients and allow early and appropriate clinical and therapeutic intervention thus reducing their increased risk of CV-related mortality [1,6,7]. ABI is recognised as a method that is accurate and reliable in the diagnosis of PAD, compared to angiography and Doppler ultrasound [8-10]. However, although ABI is easily assessable, this technique is poorly utilised in general practice and PAD is still a disease that continues to be under diagnosed and under treated [11]. Moreover, few studies are actually available with an adequate sample size to accurately describe the prevalence of low ABI in the adult population with moderate CV risk, as defined in NCEP ATP-III guidelines [7,12].

ABI has recently been evaluated for its use in improving the accuracy in prediction of CV risk in combination with the Framingham Risk Score (FRS), compared to the use of FRS alone [13]. This study demonstrated that a low ABI (≤0.90) was associated with a significant increase in the rate of CV morbidity and mortality compared with the overall rate for FRS. Due to the low prevalence of PAD within this patient group, the U.S. Preventive Services Task Force recommends against routine screening for PAD in asymptomatic individuals [14], as screening for PAD in the general population would have few or no benefits. However, current knowledge concerning the epidemiology of PAD is mainly limited to studies carried out in northern European and northern American populations [15-19], and, only more recently, 2 studies performed in southern European countries [20-22]. Recently, due to the increasing weight of evidence on the use of the ABI to predict cardiovascular morbidity and mortality, current Task Force guidelines now supports the measurement of ABI in patients at risk of PAD [23]. Since epidemiological data for atherosclerotic disease may be markedly affected by a range of factors, including genetics, ethnics, diet, lifestyle/environment etc., it is therefore also plausible that the prevalence and natural history of PAD in Southern European countries may differ from that of Northern European countries. Actually, to date, just one Italian prevalence study has been performed on the symptomatic PAD population [20]. The aim of this study was to examine the prevalence of asymptomatic PAD using ABI, in subjects with moderate risk of cardiovascular disease (CVD) in the Italian population.

## Methods

The methods and main findings from the PANDORA study have been published elsewhere [24,25]. In brief, PANDORA was a non-interventional cross sectional study performed across 6 European countries (Italy, Belgium, France, the Netherlands, Greece and Switzerland), that examined the prevalence of PAD in subjects (N = 9816) with moderate cardiovascular risk and the absence of diabetes or overt vascular disease (NCT00689377). The present study is a post-hoc analysis performed on the Italian sub-population of the original PANDORA study [25], comprising 5112 evaluable subjects from 197 Family Medicine Investigation Centers. Inclusion criteria were men aged ≥45 years or women aged ≥55 years (CVD risk factor) and the presence of at least one of the following CVD risk factors: cigarette smoking, hypertension, diagnosed dyslipidemia, family history of early coronary heart disease (CHD) or elevated abdominal circumference. Exclusion criteria included symptoms of PAD, diabetes, established CHD or risk-equivalents and the absence of fatty serum data within the past year. The study was approved by local ethic committees, and informed consent was obtained from all participants before admission to the study.

### Study objectives

In the original study, the primary objective was to determine the prevalence of PAD, defined as an ABI score of ≤0.90, according to ACC/AHA guidelines for the management of patients with PAD [1,23,26]. Secondary outcome variables included the following: frequency of risk factors (smoking habits, family history of early CHD, hypertension, low HDL-C, high LDL-C, dyslipidemia, increased waist circumference, as defined above), and of lifestyle habits (reduced physical activity) [12,26]; treatment of risk factors; CHD risk assessed according to the Framingham Point Scores risk chart, based on sex, age, total cholesterol (TC), HDL-C, smoking habits, systolic blood pressure and treatment status data [12,27]; risk of CV death assessed according to the Systematic Coronary Risk Evaluation (SCORE) risk chart, based on sex, age, TC, smoking habits, and systolic blood pressure data [28]; association of PAD with subject and physician features as assessed by questionnaires.

### Clinical evaluation

Outpatients reported outcomes including a 14-item questionnaire on awareness of CVD risk factors, knowledge and awareness of PAD, CVD risk perception and treatment, and frequency of medical consultation was obtained in a single visit. Furthermore, a 31-items investigator questionnaire was used to investigate demographic data, and information on knowledge and behaviour in CVD diagnosis and management, quantity of CVD cases experienced, general attitudinal statements, guidelines and goals, treatment, and treatment review. Each investigator received training on ABI measurement technique, according to ACC/AHA guidelines for PAD treatment [1,23,29]. Right and left ABI measurements were performed at rest assessing the systolic blood pressure from both brachial, dorsalis pedis and posterior tibial artery, while the subject stayed in the supine position for 10 minutes. Measurements were taken with blood pressure armbands appropriately sized to the subject's lower calf (immediately above the ankle). A hand-held Doppler ultrasonography instrument Elite 100R, Nicolet Vascular Inc. (Golden, Colorado, USA), with a 8 MHz vascular probe, was used in all the centres. Right and left ABI values were calculated according to ACC/AHA guidelines [1,23,29]. The primary outcome variable was the percentage of subjects with PAD, defined as a pathological ABI of ≤0.90 measured from the left or right side. The original PANDORA study was performed according to guidelines laid down in the Declaration of Helsinki, are consistent with ICH/Good Clinical Practice, applicable regulatory requirements and the AstraZeneca policy on Bioethics.

### Sample size and statistical analysis

Statistical analysis was performed with SAS version 8.2 software (SAS Institute Inc., Cary, NC, USA). Data are presented as mean ± SD and/or range and frequency was expressed as a percentage. Groups were compared using the χ^2 ^test. For the original PANDORA study [25], the frequency (%) of the study endpoint with a two-sided 99% confidence interval (CI) of ≤1% was determined. Based on an expected 15% frequency of pathological ABI in the selected population [30], a minimum sample size of 8, 454 evaluable subjects was required to be able to reach study objectives, and a total population of 9, 000 subjects was therefore planned to be admitted. To examine the relationships between abnormal ABI and lifestyle habits or cardiovascular risk factors, the Odds ratio (OR) and 95% CI of frequency of risk factors and lifestyle habits in subjects with ABI ≤0.90 and ABI > 0.90 were calculated and differences between groups were tested by the Cochran-Mantel-Haenszel test. The relationship between PAD (defined as presence of ABI ≤0.90) and the features of patients admitted and family doctors participants was assessed by logistic regression with backward selection of covariates, with calculation of OR and 95% CI. Subject data were investigated among the following variables: country, demographics (age, sex, ethnic origin, marital status), CVD risk factors and lifestyle habits (smoking habits, alcohol intake, physical activity, family history of early CHD, hypertension, diagnosed dyslipidemia, waist circumference, high LDL-C, low HDL-C), and treatment with lipid-lowering drugs. The primary analysis was performed on evaluable subjects. All subjects admitted to the study signed informed consent, had no protocol violations, had an ABI measurement and > 80% of data collected in subject record forms. A value of p < 0.05 was considered statistically significant; N values refer to the number of patients examined.

## Results

From a total of 10, 287 subjects admitted to the original PANDORA study, 5, 298 of these subjects were enrolled in Italy (51.6% of total) from May 2007 to July 2008. Among admitted subjects, 96.5% (N = 5, 112) were considered evaluable. The most frequent reasons for exclusion were: a) less than 80% of CRF fields completed (2.45%), b) unmeasurable ABI (2.36%) and c) a lack of serum lipid data collected within the previous 12 months (0.68%).

Baseline demographic and clinical characteristics for evaluable subjects with and without PAD are summarized in Table 1. Both groups of patients with and without PAD were well matched for age and gender. Almost all subjects were Caucasian (99.51%), approximately half were male gender (50.69%) and the mean age was 64 years. Significant differences were observed between subjects with and without PAD for several parameters measured. PAD subjects had significantly increased blood pressure (SBP and DBP, p < 0.0001) and heart rate (p < 0.0001). Furthermore, the proportion of patients with hypertension, family history of early CHD and high alcohol intake was significantly higher in PAD subjects. Serum lipids and lipid modifier medication (including statins) were also significantly different between subjects with and without PAD. Interestingly, a significant lower proportion of patients with PAD were married or living together compared to patients without PAD (68% versus 79.7%. p < 0.0001). Likewise, the percentage of subjects separated or divorced were significantly higher in the PAD group (no PAD compared to PAD respectively; 3.91% versus 13.94%, p < 0.0001).

**Table 1:** Demographic and clinical characteristics of evaluable subjects with and without PAD.

Clinical characteristics	No PAD (N = 3, 943)	PAD (N = 1, 169)	P value
Weight (Kg)	76.5 ± 14	77.1 ± 14.3	
Waist circumference (cm)	98 ± 12	97.9 ± 12.8	
BMI (Kg/M^2^)	27.8 ± 4.3	27.8 ± 4.5	
SBP (mmHg)	136.4 ± 14.6	141 ± 14.6	< 0.0001
DBP (mmHg)	81.6 ± 8.3	83 ± 8.2	< 0.0001
Heart rate (beats/min)	72.8 ± 8.7	74.2 ± 8.2	< 0.0001
Age (years, mean ± SD)	63.9 ± 8.7	64.1 ± 9.2	
Median	63	64	
Interquartile range	58-70	58-70	
Age class (%)			
45-54 years	11.4	12.8	
55-64 years	44	41	
65-74 years	32.3	32.1	
75-84 years	11.2	12.2	
≥85 years	1.1	1.9	0.039
Sex			
Male	50.4	51	
Female	49.6	49	
Race (%)			
Caucasian	99.5	99.6	
Black	0.1	0.1	
Oriental	0	0	
Other	0.4	0.3	
Marital status (%)			
Single (unmarried)	4.9	4.5	
Married or living together	79.7	68	< 0.0001
Widowed	11.4	13.5	
Divorced or separated	3.9	13.9	< 0.0001
Other	0.1	0.1	
Anamnesis (%)			
Hypertension	73.7	77.3	0.016
Dyslipidemia	45.2	37.6	< 0.0001
Cigarette smoking	30.2	25.2	0.0008
High alcohol intake	6.1	8.1	0.015
Family history of early CHD	17.4	41.8	< 0.0001
Sedentary lifestyle	64.7	61.2	
Serum lipids (mg/dl)			
Total cholesterol	216.3 ± 39.2	216.9 ± 38.3	
HDL-cholesterol	53.7 ± 13.4	51.9 ± 12.9	< 0.001
LDL-cholesterol	134.4 ± 35.4	134.8 ± 35.4	
Triglycerides	138.6 ± 62.7	156.4 ± 70	< 0.0001
Medication (%)			
Antihypertensive	54.5	60.05	0.003
Other CV drug	7.23	5.82	
Any lipid modifiers	18.18	14.29	0.002
Statin treatment	11.6	6.5	< 0.0001

Data are presented as mean ± SD or % where indicated. BMI = body mass index, CHD = coronary heart disease, SBP = systolic blood pressure, DBP = diastolic blood pressure.

**Figure 1:**
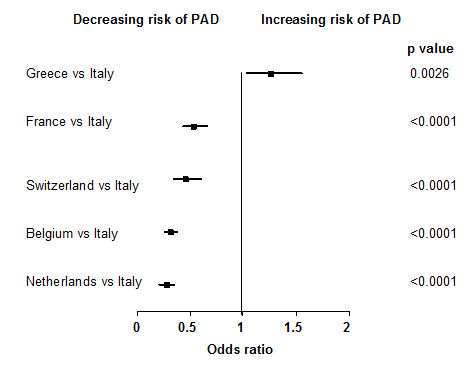
**Comparison of risk of PAD between other European countries compared to Italy**. Multiple logistic regression model of statistically significant regressors. Data presented as odds ratio (upper and lower 95% confidence intervals). P values refer to level of statistical significance. Data derived from original PANDORA study (N = 9816).

Among the 6 participating countries of the previous PANDORA study, a higher prevalence of PAD was observed in Greece (28.0%) and Italy (22.9%), rather than in France (12.2%),

Belgium (7.0%), the Netherlands (8.1%) or Switzerland (12.2%). Multiple logistic regression analysis confirmed that after Greek subjects, Italians had a greater risk of PAD (Figure 1).

**Figure 2:**
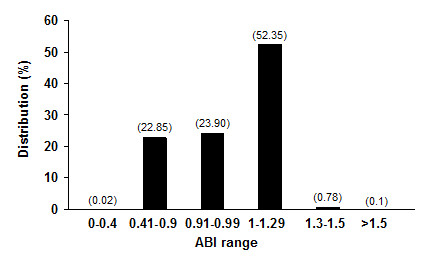
**Distribution of ABI in Italian subjects**. Data are presented as percentage distribution, actual values are represented in parentheses.

Distribution of ABI values in all Italian subjects is shown in Figure 2. Overall, 22.9% of subjects had an ABI ≤0.9 and another 23.9% between 0.91-0.99. Approximately half of subjects observed had an ABI > 0.99 (53.23%).

**Figure 3:**
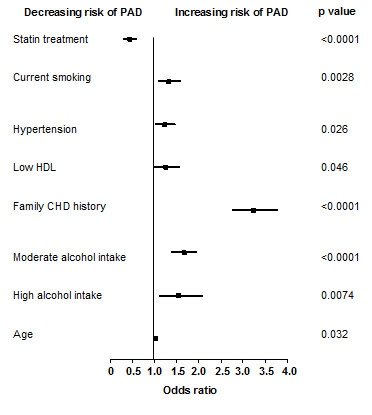
**The association between PAD and number of individual CVD risk factors**. Multiple logistic regression model of statistically significant regressors. Data presented as odds ratio (upper and lower 95% confidence intervals). P values refer to level of statistical significance. Data based on Italian sub-population from original PANDORA study (N = 5112).

We next evaluated the relationship between PAD and individual subjects determinants (country, age, sex, race, marital status), lifestyle habits (alcohol intake, physical activity), and CVD risk factors (smoking, family history of early CHD, hypertension, elevated waist circumference, dyslipidemia diagnosis and treatment, high LDL-C, low HDL-C, statin treatment). Multiple logistic regression did not yield any difference regarding gender, race, physical activity, elevated waist circumference, dyslipidemia, high LDL-C levels or BMI. Variables significantly associated with PAD are shown in Figure 3

To examine the cumulative impact of CVD risk factors on presence or absence of PAD, summary statistics of the 10-year CHD risk were calculated according to the Framingham risk score (Table 2). The Framingham 10-year CHD risk score was higher in subjects with PAD compared to those without PAD, in terms of both mean and median values and risk frequency distribution. PAD subjects also showed a higher frequency in the high-risk class (> 20%) compared to subjects without PAD (23.95% PAD versus 15.98% without PAD).

**Table 2:** Summary statistics of 10-year CHD risk - according to Framingham algorithm in the Italian population with and without PAD

CHD risk		No PAD (N = 3, 943)	PAD (N = 1, 169)
	Mean ± SD	12.98 ± 8.10	14.68 ± 8.49
Risk score	Median	12	14
	Interquartile range	0-30	1-30
			
	< 10%	38.68	32.85
Risk class	10-20%	45.35	43.2
	> 20%	15.98	23.95

**Figure 4:**
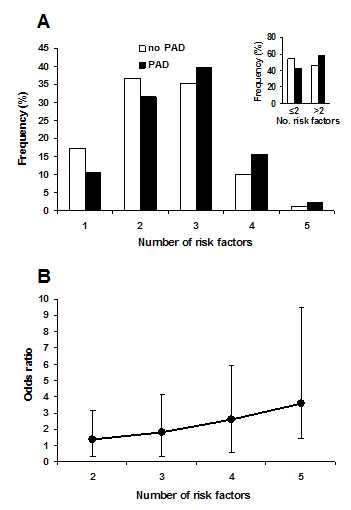
**The association between PAD and the number of CVD risk factors**. A, The frequency of CVD risk factors (1-5 risk factors) is shown for patients with no PAD (open bars) and patients with PAD (filled bars). Inset, % patients (presence or absence of PAD) with ≤2 or > 2 risk factors. B, Association between risk of PAD and the number of CVD risk factors in addition to age. Data expressed as Odds ratio (± 95% CI). Comparisons were made between estimates of frequency distribution of the number of risk factors (i.e. ≥2 risk factors vs 1 risk factor) by Cochran-Mantel-Haenszel test.

To explore risk factor burden, the cumulative number of CVD risk factors in each subject was analysed. In addition to age, most subjects (with or without PAD) presented 2 or 3 CVD risk factors (Figure 4A). Whenever subjects were evaluated for the presence of ≤2 or > 2 risk factors, the frequency of PAD was higher with the presence of 2 risk factors (Figure 4A, inset). Therefore, the frequency of subjects with PAD and without PAD was greatest in the SCORE 10-year CV death risk class (> 2%) (Table 3). However, frequency of PAD subjects was higher than subjects without PAD for high-risk classes (3-4%, 5-9% and 10-14%).

**Table 3:** Summary statistics of 10-year CV risk - according to SCORE algorithm in the Italian population with and without PAD.

Risk class	No PAD (N = 3, 943)	PAD (N = 1, 169)
< 1%	192 (4.9)	40 (3.4)
1%	1007 (25.5)	250 (21.4)
2%	1033 (26.2)	299(25.6)
3-4%	853 (21.6)	274 (23.4)
5-9%	754 (19.1)	248 (21.2)
10-14%	79 (2)	51 (4.4)
≥15%	25 (0.63)	7 (0.6)

Data presented as number and (%).

The association between PAD and the number of CVD risk factors (in addition to age), expressed in terms of OR ± 95% CI estimates of frequency of ≥2 risk factors compared to 1 risk factor is shown in Figure 4B. Subjects presenting ≥2 CVD risk factors in addition to age (i.e. ≥3 CVD risk factors) are more affected by PAD compared to subjects with only 1 additional risk factor (p < 0.0001, Cochran-Mantel-Haenszel test).

Concomitant use of other cardiovascular drugs was similar in subjects with PAD than in those without PAD (5.82% vs 7.23%). The quantity of subjects receiving anti-hypertensive drugs was also similar in both groups (54.5% vs 60.05%). However, statin therapy was significantly associated with absence of PAD (Figure 3).

## Discussion

This post-hoc analysis of the PANDORA Study represents the largest study on the general Italian population to date that has focussed on the prevalence of asymptomatic PAD in the community setting. Previously, another study investigated the prevalence of PAD in Italian subjects, but only patients with symptomatic PAD were evaluated [20]. The present study is in agreement with findings from the original PANDORA study since it confirms the prevalence of a low ABI in patients who would otherwise be classified as intermediate and even low CV risk [25]. The prevalence of asymptomatic PAD within the Italian population was 22.9%. Limited use of statins in the total cohort of Italian subjects (10.5%) was also confirmed within the subgroup diagnosed with dyslipidemia (24%). Other participant countries had a higher percentage of patients on statins. The risk of developing PAD is also increased in subjects with a higher consumption of saturated fatty acids [31], which is common in northern Europe [32]. In contrast, risk is reduced in subjects with higher intake of cereal fiber, Vitamin E and wine consumption [31,33,34], which are typical components in the so-called "Mediterranean diet" [32]. These food preferences in Italian people may have a favourable action to decrease cardiovascular risk for PAD. Although gender was equally matched in the present study, this may be a potential factor to consider when comparing across differences in other European countries. However, two separate studies, performed on a Turkish and a US population respectively, have demonstrated that the prevalence of PAD does not vary by gender [22,35]. Subjects without symptoms of the lower limbs and overt CV disease who are at risk of PAD, including those less than 50 years with diabetes and one additional risk factor (smoking, dyslipidemia, hypertension, or hyperhomocysteinemia), or aged 50 to 69 with a history of smoking or diabetes, or aged 70 and older need to be further screened for PAD

as highlighted in the PARTNERS study [19]. These patients need to be submitted to clinical, diagnostic and instrumental assessment, according to recommendations mainly derived from the PARTNERS study [19]. Another ABI study performed in Germany on 6, 880 unselected outpatients in Primary Care, aged ≥65 years, who underwent ABI testing by family physicians in 344 centres, showed a prevalence of PAD of 18% [36,37]. This frequency is in agreement with results of the present study, even though in the Get ABI study 2.8% of the subjects with low ABI had PAD symptoms. In addition, risk profile in the Get ABI population showed differences from that of PANDORA, which was due to fewer previous cardiovascular events or the presence of diabetes. Nevertheless, as evidenced by PANDORA, the Get ABI also confirmed the usefulness of expanding measurement of ABI over to other risk categories beyond those indicated by guidelines. A more recent study performed in Turkey showed a 20% prevalence of PAD, corroborating findings observed in our study [22]. Moreover, demographic and clinical characteristics of this Turkish population were similar to the Italian cohort (i.e., age, gender ratio, associated risk factors, etc.), suggesting that a relatively high prevalence of PAD can be observed in other Mediterranean-like lifestyles. Previous data reported from the National Health and Nutrition Examination Study (NHANES; 1999-2004) showed a 3% prevalence of low ABI in the low or intermediate risk population [38]. Furthermore, Sumner et al. analyzed data from three NHANES between the years 1999 -2004 and showed that the prevalence of PAD is consistently increasing in asymptomatic adults in the US population. Prevalence rose from 3.7 to 4.6% (p = 0.001) over a six year period [39]. Marked differences between these results and those from the PANDORA study were observed, where even in countries with minimal prevalence of low ABI, frequencies of over 3% were reported, thus emphasizing the need for periodical measurement of ABI in many risk-groups. There are some notable findings resulting from this study, such as the relationship between restricted use of statins and the prevalence of low ABI. The well recognised beneficial prognostic effect of statin treatment on the prevention of vascular events in patients with symptomatic PAD [40] should be emphasized, with the view to improving their lower extremity motor performance [41]. Our findings from the PANDORA study show a possible protective action of statins against the occurrence of PAD and may assist in re-evaluating the pathogenetic role of dyslipidemia in this condition, especially considering the fact that other characteristics normally altered by statins, such as inflammation, were not taken into account.

It is also worth highlighting the association between marital status and the presence of low ABI, with unmarried or widowed subjects more likely affected by low ABI than married subjects. This shows unexpected evidence on the relationship between marital status and prevalence of PAD, although previous reports have described the role of marital status, as part of a more general social support, as influencing clinical symptoms of PAD [42]. Although we have not directly examined what underlying factors may influence the link between marital status (divorced/separated/unmarried) and prevalence of PAD, it is recognised that the rate of depressive symptoms is increased in these individuals [43,44]. It is well documented that patients with PAD have a higher rate of depression and that this is also correlated with the reduced activity/sedentary lifestyle that they lead [45]. However, our findings did not reveal any discernible difference in sedentary lifestyle between patients with or without PAD, suggesting that this cause-and-effect relationship between marital status and prevalence of PAD is more complex.

The major interest of psycho-social factors as cardiovascular risk factors is also suggested by these PANDORA findings [46]. The explanation for the different frequency distribution of PAD among countries may be related to the variability of subject characteristics, in terms of both frequency distribution and type of risk factors, and could have been influenced by several lifestyle habits [20] in addition to differences in use of statins between several countries.

This study has some limitations, including the lack of a follow-up period on the occurrence of cardiovascular events, over an appropriate period after ABI assessment, in subjects presenting with a low ABI but otherwise classified as being at low or moderate risk and, as such, not considered candidates for more aggressive preventive treatment. However, the adverse prognostic implications of a low ABI (≤0.90) are widely known and are derived from many previous studies that have definitively confirmed this instrumental examination as an independent predictor of high cardiovascular risk [2,4]. Therefore, the standard use of Doppler remains indispensable for an adequate ABI measurement in family practice, as reported in healthy subjects, patients at risk and patients clinically suspected for PAD [47]. It should be emphasized that most recent epidemiological studies on PAD have been performed only [21] or almost only [30] in primary care service, placing ABI measurement in the daily clinical practice of the general practitioner. The ABI value may be limited in some patients because of the calcification of tibial arteries that are less compressible, resulting in unusually high ABI values (> 1.40) [1]. Data from a recent study by Cacoub and coworkers [30] in a subset of 2, 077 patients older than 55 years and with 2 or more cardiovascular risk factors (including diabetes) in which the ABI was measured by general practitioners in France, provided a prevalence of low ABI (below 0.90) of 10.4%, which is similar to that of the French population of the PANDORA study, confirming the satisfactory reliability of the data collected [25]. The results reported in the present study are in agreement to other previous studies in the general population [22,36,37]. The prevalence of PAD evaluated using the ABI was found to be 18-19% in subjects aged above 55 or 65 years in The Netherlands, Germany, the UK, and at approximately 30% in patients with selected vascular risk factors in North America and France [18,19,48-50]. Given the very high prevalence of PAD, we observed in moderate-risk patients and the CV morbidity and mortality associated with PAD [1,2], undoubtedly better public and health professional awareness would help preserve individual CV health and achieve public health goals.

## Conclusions

This study provides further evidence that a low ABI is significantly associated with classical CV risk factors. Compared to other countries (from the original PANDORA study) this Italian population showed a restricted statin medical prescription and treatment, although it is unlikely that this effect may alone account for this increased prevalence. This study indicates the appropriate use of ABI in Family Medicine for the identification of patients at high risk and therefore allowing early treatment for the reduction of CV-related death.

## Abbreviations

ABI: ankle-brachial index; CHD: coronary heart disease; CI: confidence interval; CV: cardiovascular; CVD: cardiovascular disease; FRS: Framingham Risk Score; HDL: high-density lipoprotein; LDL: low-density lipoprotein; PAD: peripheral arterial disease; PANDORA: Prevalence of peripheral Arterial disease in patients with a non-high CVD risk, with No overt vascular Diseases nOR diAbetes mellitus.

## Competing interests

GS has been Italian Coordinator of the PANDORA study, and as consultant on PAD Advisory Board and has no financial or non-financial competing interests. DA has been Italian Coordinator of the PANDORA study, and as consultant on PAD Advisory Board and has no financial or non-financial competing interests. MM has been Italian Coordinator of the PANDORA study, and as consultant on PAD Advisory Board and has no financial or non-financial competing interests. CC has no financial or non-financial competing interests. He has acted as a speaker and chairman at scientific meetings sponsored by AstraZeneca, Sanofi-Aventis and Bristol-Myers Squibb. CB has acted as a consultant or speaker on occasions for Recordati, AstraZeneca, Merck, MSD, Novartis, Boehringer Ingelheim, Takeda, and Schering-Plough, and has received research funding from Boehringer Ingelheim, Sanofi-Aventis, Takeda, Italian Society of Hypertension, and the

Regional Drug Agency. He holds shares in Abbott and Bristol-Myers Squibb and has received honoraria as a speaker in international and national meetings. He does not have any conflict of interest relating to the present activity. ALF has been Italian Coordinator of the PANDORA study, and as consultant on PAD Advisory Board and has no financial or non-financial competing interests. MM is an employee of AstraZeneca

## Authors' contributions

GS participated in the study design, evaluated the statistical analysis, and drafted the manuscript. DA participated in the study design, evaluated the statistical analysis, and drafted the manuscript. MM^1 ^participated in the study design and drafted the manuscript. CC participated in the study design, evaluated the statistical analysis, and drafted the manuscript. CB participated in the study design, evaluated the statistical analysis and drafted the manuscript. ALF participated in the study design and drafted the manuscript. MM^2 ^participated in the study design, evaluated the statistical analysis, and drafted the manuscript. All authors read and approved the final manuscript.

## Pre-publication history

The pre-publication history for this paper can be accessed here:

http://www.biomedcentral.com/1471-2261/11/59/prepub
